# Comparative Transcriptome Analysis of the Hypothalamic–Pituitary–Gonadal Axis of Jinhu Grouper (*Epinephelus fuscoguttatus* ♀ × *Epinephelus tukula* ♂) and Tiger Grouper (*Epinephelus fuscoguttatus*)

**DOI:** 10.3390/genes15070929

**Published:** 2024-07-16

**Authors:** Yishu Qiu, Pengfei Duan, Xiaoyu Ding, Zhentong Li, Xinyi Wang, Linlin Li, Yang Liu, Linna Wang, Yongsheng Tian

**Affiliations:** 1 State Key Laboratory of Mariculture Biobreeding and Sustainable Goods, Yellow Sea Fisheries Research Institute, Chinese Academy of Fishery Sciences, Qingdao 266071, China; yishuqiu97@163.com (Y.Q.);; 2 Laboratory for Marine Fisheries Science and Food Production Processes, Qingdao Marine Science and Technology Center, Qingdao 266237, China; 3 Hainan Innovation Research Institute, Chinese Academy of Fishery Sciences, Sanya 572000, China

**Keywords:** Jinhu grouper (*Epinephelus fuscoguttatus* ♀ × *Epinephelus tukula* ♂), *Epinephelus fuscoguttatus*, gonadal development, transcriptome

## Abstract

Jinhu groupers, the hybrid offspring of tiger groupers (*Epinephelus fuscoguttatus*) and potato groupers (*Epinephelus tukula*), have excellent heterosis in fast growth and strong stress resistance. However, compared with the maternal tiger grouper, Jinhu groupers show delayed gonadal development. To explore the interspecific difference in gonadal development, we compared the transcriptomes of brain, pituitary, and gonadal tissues between Jinhu groupers and tiger groupers at 24-months old. In total, 3034 differentially expressed genes (DEGs) were obtained. KEGG (Kyoto Encyclopedia of Genes and Genomes) enrichment analyses showed that the osteoclast differentiation, oocyte meiosis, and ovarian steroidogenesis may be involved in the difference in gonadal development. Trend analysis showed that the DEGs were mainly related to signal transduction and cell growth and death. Additionally, differences in expression levels of *nr4a1*, *pgr*, *dmrta2*, *tbx19*, and *cyp19a1* may be related to gonadal retardation in Jinhu groupers. A weighted gene co-expression network analysis revealed three modules (i.e., saddlebrown, paleturquoise, and greenyellow) that were significantly related to gonadal development in the brain, pituitary, and gonadal tissues, respectively, of Jinhu groupers and tiger groupers. Network diagrams of the target modules were constructed and the respective hub genes were determined (i.e., *cdh6*, *col18a1*, and *hat1*). This study provides additional insight into the molecular mechanism underlying ovarian stunting in grouper hybrids.

## 1. Introduction

Distant hybridization (DH) is widely used in fish breeding and can have significant economic benefits. DH can combine the beneficial traits of the parents to obtain hybrid offspring with heterosis and can effectively resolve low fecundity, low disease resistance, population degradation, and other issues caused by long-term inbreeding [1]. Trait values in hybrid offspring may surpass those of the parents in some cases [2], for example, hybrid offspring can grow faster [3] and have stronger stress resistance [4].

The grouper is an important economic fish in the world because of its excellent traits. At present, the grouper industry faces challenges related to a reduction in natural resources, lack of breeding varieties, low survival rate of fingerlings, and low stress resistance [5]. The breeding cycle is long and selection efficiency is low. Hybrid breeding can quickly combine beneficial traits of parents to produce excellent hybrid offspring. Therefore, many attempts have been made to hybridize groupers, such as *Epinephelus fuscoguttatus*♀ × *Epinephelus lanceolatus*♂ [6], *Epinephelus coioides*♀ × *E. lanceolatus*♂ [7], *Epinephelus moara*♀ × *E. lanceolatus*♂ [8], and *E. fuscoguttatus*♀ × *Epinephelus polyphekadion*♂ [9].

Using frozen sperm of the potato grouper (*E. tukula*) and eggs of the tiger grouper (*E. fuscoguttatus*), we obtained the hybrid offspring “Jinhu grouper” with excellent heterosis [10]. Our team has studied the Jinhu grouper from the aspects of morphology [11], genetics [12,13], and stress resistance [14,15,16]. The gonadal development and fertility of hybrids in culture and production systems have been raised as concerns by some scholars. The potential for introgression between hybrids and natural populations is another concern. We have previously found that the Jinhu grouper shows gonadal growth retardation compared with growth in the tiger grouper [17]. In this study, the molecular mechanism underlying gonadal retardation in hybrids was further explored.

Fish reproduction is regulated by the hypothalamic–pituitary–gonadal (HPG) axis and is influenced by various environmental factors. Briefly, the central nervous system transduces environmental factors and social behaviors to regulate the synthesis and release of gonadotropin-releasing hormone (GnRH), gonadotropins (GtHs), and sex steroid hormones [18]. Many pathways and related genes participate in this process, including the MAPK signaling pathway [19], p53 signaling pathway [20], and oocyte meiosis [21]. Previous studies of gonadal development of hybrid groupers have primarily focused on phenotypic and structural observations [22,23], determination of sex steroid hormones [24], and comparisons of diploid and triploid gonadal development [25]. For a deeper understanding of the reproductive mechanism of the Jinhu grouper, it is necessary to obtain a transcriptome map for the whole HPG axis.

In this study, brain, pituitary, and gonadal tissues of Jinhu groupers and tiger groupers were sequenced. The mRNA expression patterns of these two grouper types were analyzed to study the changes in gene expression of gonadal retardation in the Jinhu grouper, laying a foundation for better understanding the reproductive mechanism of the hybrid.

## 2. Materials and Methods

### 2.1. Ethics Statement

All animal experiments were approved by the Yellow Sea Fisheries Research Institute’s Animal Care and Use Committee (No. YSFRI-2024056).

### 2.2. Fish and Sampling

In March 2020, three families of Jinhu grouper (1249 individuals) and one family of tiger grouper (929 individuals) were constructed. These families were cultured in the Laizhou Ming Bo Aquatic Co., Ltd. (Yantai, China). The industrial culture conditions were as follows: water volume 40 m^3^, water temperature 21–26 °C, dissolved oxygen 6–9 mg/L, salinity 28–30‰, and artificial formula feed (Santong Bioengineering Co., Ltd., Weifang, China). Feeding was twice a day during the seedling period, and the specifications and amounts of feed varied with the growth status of the fish, and once a day in the adult period (2–3% of the fish body weight).

Under the same culture conditions, three healthy Jinhu groupers and three healthy tiger groupers were selected at the age of 24 months. The body lengths of the Jinhu groupers and tiger groupers were 37.47 ± 0.65 cm and 28.53 ± 1.19 cm, respectively. The brains, pituitaries, and gonads were dissected out after anesthesia with 180 mg/L MS-222 (Sigma, Shanghai, China) and stored at −80 °C for RNA extraction.

### 2.3. RNA Extraction, Library Construction, and Sequencing

RNA extraction, library construction, and sequencing were performed according to Duan et al. [16]. Briefly, TRIzol reagent (Invitrogen, Carlsbad, CA, USA) was used for the extraction of total RNA for each sample. An Agilent 2100 bioanalyzer (Agilent Technologies, Santa Clara, CA, USA) and RNase-free agarose gel were used for the verification of RNA quality. The samples with an RNA integrity number higher than 7 were used for cDNA library construction. Eighteen cDNA libraries were sequenced using Illumina NovaSeq 6000 by Gene Denovo Biotechnology Co., (Guangzhou, China). The raw reads were deposited in the National Center for Biotechnology Information (NCBI, https://www.ncbi.nlm.nih.gov/) (accessed on 13 April 2023) (accession number PRJNA955319).

### 2.4. Analysis of Differentially Expressed Genes (DEGs)

Differentially expressed gene (DEG) analyses were performed according to Duan et al. [16]. In this study, false discovery rate (FDR) < 0.05 and absolute value of log_2_ (|log_2_(FC)|) > 1 were used as thresholds to screen DEGs. All DEGs were mapped to the Gene Ontology database (GO, http://www.geneontology.org/) (accessed on 17 October 2023) and Kyoto Encyclopedia of Genes and Genomes database (KEGG, http://www.genome.jp/kegg/) (accessed on 11 November 2023) to further understand the genes’ biological functions. The GO terms and KEGG pathways that met *p* < 0.05 were defined as biologically significant.

### 2.5. Trend Analysis

In order to explore the regulatory mechanism of the HPG axis on reproduction, in this study, a trend analysis was carried out along the HPG axis according to Duan et al. [16]. All the DEG files of the grouped samples were input sequentially, and then the trend analysis was carried out, and *p* < 0.05 was taken as the significant profiles’ screening threshold. Then, functional enrichment analyses were performed on the DEGs in each profile.

### 2.6. Weighted Gene Co-Expression Network Construction

A weighted gene co-expression network analysis (WGCNA) was performed according to Duan et al. [16]. After filtering genes with a FPKM (fragment per kilobase of transcript per million mapped reads) value < 1, the remaining high-quality genes were imported into WGCNA to construct co-expression modules. The genes with similar expression patterns were clustered using R package of WGCNA (v1.47), and the correlation coefficients between modules and samples were analyzed to identify biologically significant modules. The intramodular connectivity of each gene was calculated, and the gene with the highest connectivity was determined as the hub gene. Functional enrichment analyses of the genes in each module were performed. Finally, networks for the visualization of hub genes associated with gene interactions were constructed using Cytoscape v3.9.1 (https://cytoscape.org/) (accessed on 21 January 2024).

### 2.7. Experimental Validation with Quantitative Real-Time PCR (qRT-PCR)

The validation of the RNA sequencing results was performed by quantitative real-time PCR (qRT-PCR) according to Duan et al. [16]. Four genes (*fez2*, *alas2*, *nmrk2*, and *apoa1*) in the brain, three genes (*mtap*, *hoxa2a*, and *actb*) in the pituitary, and five genes (*zp4*, *jun*, *igfbp1*, *h1-0-b*, and *gstf14*) in the gonad were selected for qRT-PCR validation. These genes were the ones found to have large fold differences during the analysis. Specific primers for 12 DEGs were designed using Primer Premier 5 (Premier Biosoft, San Francisco, CA, USA) (Table S1). The remaining RNA samples from transcriptome sequencing were reverse-transcribed to cDNA. β-actin was used as the reference gene [26]. Relative expression levels of the DEGs were calculated using the 2^−ΔΔCt^ method, and are expressed as means ± standard deviation (SD) of triplicates.

## 3. Results

### 3.1. Transcriptome Overview

Eighteen cDNA libraries were constructed and sequenced from the brains (B), pituitaries (P), and gonads (G) of 24-month-old Jinhu groupers (EFET) and tiger groupers (EF) (referred to as EFET_24B1-3, EFET_24P1-3, EFET_24G1-3, EF_24B1-3, EF_24P1-3, and EF_24G1-3). We obtained 479,069,804 and 396,386,758 raw reads from the Jinhu groupers and tiger groupers, respectively. The adaptors and low-quality sequences were eliminated, resulting in 477,081,996 and 394,697,426 clean reads, with Q20 and Q30 values exceeding 96.30% and 90.46%, respectively (Tables S2 and S3). The rate of the clean reads alignment to the genome of the tiger grouper was 80.38–96.62% (Table S4), indicating that the transcriptome of the tested species was highly similar to the reference genome.

### 3.2. Sample Correlations and Identification of the DEGs

**Figure 1:**
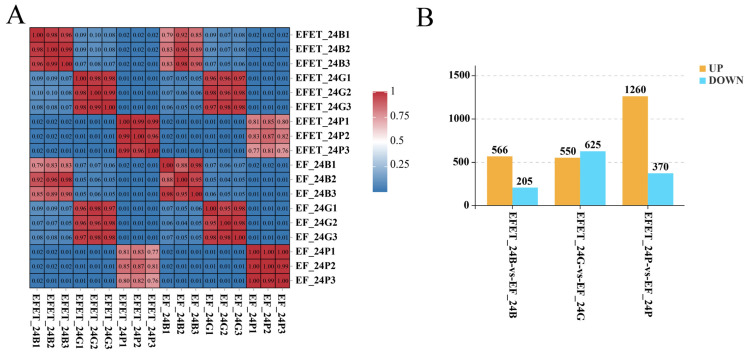
Sample correlations and summary of differentially expressed genes (DEGs) in the brain, pituitary, and gonadal tissues of the tiger grouper and Jinhu grouper at 24 months old. (**A**) Heatmap of sample correlations; (**B**) Number of DEGs in the Jinhu grouper (EFET) vs. tiger grouper (EF); yellow and blue indicate up-regulated and down-regulated genes.

For all pairwise comparisons between samples for each tissue type, the Pearson correlation coefficients between expression levels were calculated. The Pearson correlation coefficients, visualized in the form of a heatmap, were greater than 88%, revealing high consistency within the same tissue type (Figure 1A).

To identify DEGs between the Jinhu groupers and tiger groupers, we compared the transcriptomes of brain, pituitary, and gonadal tissues from the two grouper types. As shown in Figure 1B, we obtained 771 (566 up-regulated and 205 down-regulated in the brain), 1175 (1260 up-regulated and 370 down-regulated in the pituitary), 1630 (550 up-regulated and 625 down-regulated in the gonad) DEGs.

### 3.3. Functional Enrichment Analysis of DEGs

In order to further understand the biological process involved in the DEGs in the HPG axis between the Jinhu grouper and tiger grouper, a GO enrichment analysis was carried out. In total, 953, 1962, and 597 terms were identified in the brain, pituitary, and gonad, respectively (*p* < 0.05). The number of up-regulated genes in each pathway was markedly higher in the brain and pituitary compared with the number of down-regulated genes. In contrast, there were more down-regulated genes in most pathways in the gonad (Figure S1).

In the brain, we obtained 27 significantly enriched pathways (*p* < 0.05), including ovarian steroidogenesis, the JAK-STAT signaling pathway, and osteoclast differentiation. In the pituitary, we obtained 52 significantly enriched pathways (*p* < 0.05). DEGs were involved in the cell adhesion molecules, oocyte meiosis, and cholesterol metabolism. In the gonad, we obtained 36 significantly enriched pathways (*p* < 0.05). ECM-receptor interaction, metabolic pathways, and the estrogen signaling pathway were significantly enriched (Figure S2).

**Figure 2:**
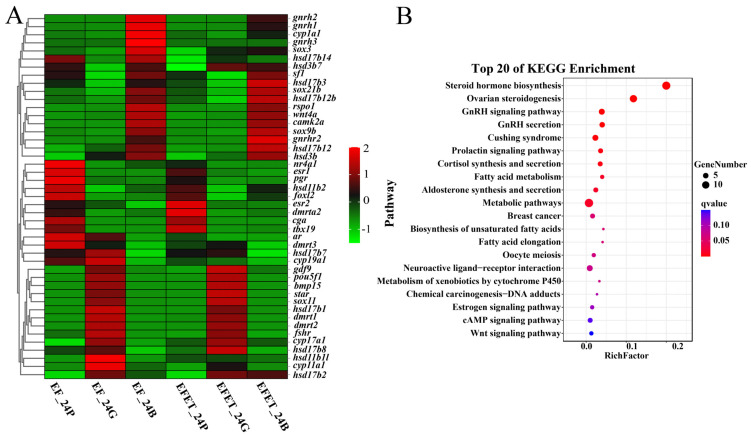
(**A**) The expression patterns of reproduction−related differentially expressed genes (DEGs) in the two grouper types. Red: higher expression, green: lower expression. (**B**) Kyoto Encyclopedia of Genes and Genomes (KEGG) enrichment of reproduction−related genes.

To explore the expression pattern of reproduction-related genes in the two grouper types, 45 known reproduction-related genes were analyzed (Figure 2A). These genes were significantly associated with lipid metabolism, the endocrine system, and global and overview maps (*q* < 0.05) (Figure 2B).

### 3.4. Trend Analysis of DEGs

**Figure 3:**
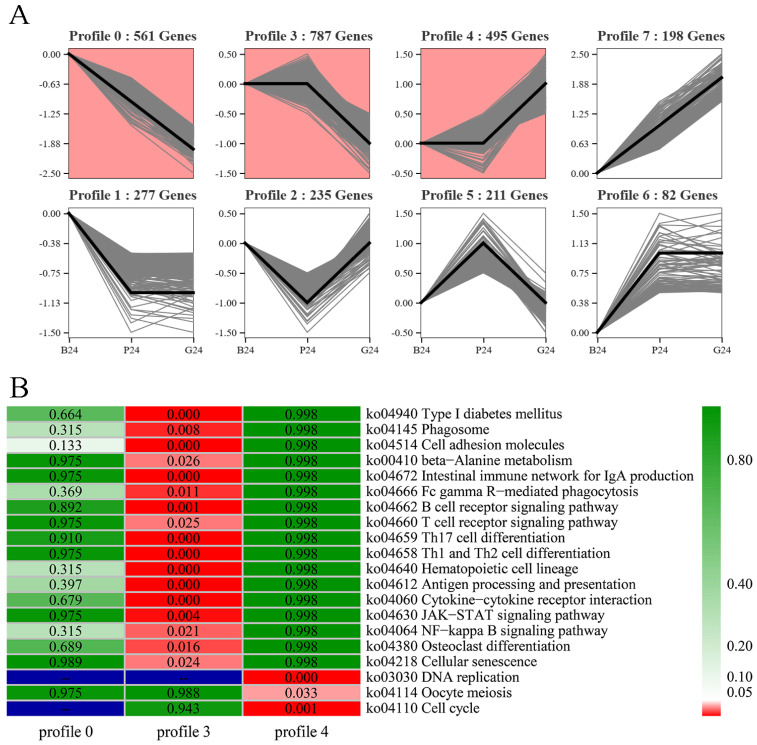
Profiles of gene expression patterns based on trend analysis and Kyoto Encyclopedia of Genes and Genomes (KEGG) enrichment analysis of significant profiles. (**A**) Eight expression profiles and the number of genes (pink profiles were statistically significant) (*p* < 0.05). The gray line in each module is the change trend of each gene expression in the module, and the black line is the overall change trend of all gene expression in the module.; (**B**) KEGG pathway enrichment analysis of significant profiles (*q*−value).

Through trend analysis, all genes could be clustered into eight profiles (i.e., profiles 0–7), of which three profiles were significant (i.e., profiles 0, 3, and 4) (*p* < 0.05) (Figure 3A). All 561 DEGs were consistently down-regulated in profile 0, and significantly enriched pathways with *p* < 0.05 were cell adhesion molecules, axon regeneration, and hematopoietic cell lineage (Figure S3). DEGs in the other significant profiles (i.e., profiles 3 and 4) were involved in the interaction between pituitary and gonad, and were enriched in the NF-κB signaling pathway, cytokine–cytokine receptor interaction, and oocyte meiosis (*q* < 0.05) (Figure 3B).

### 3.5. Construction of a Gene Co-Expression Network

**Figure 4:**
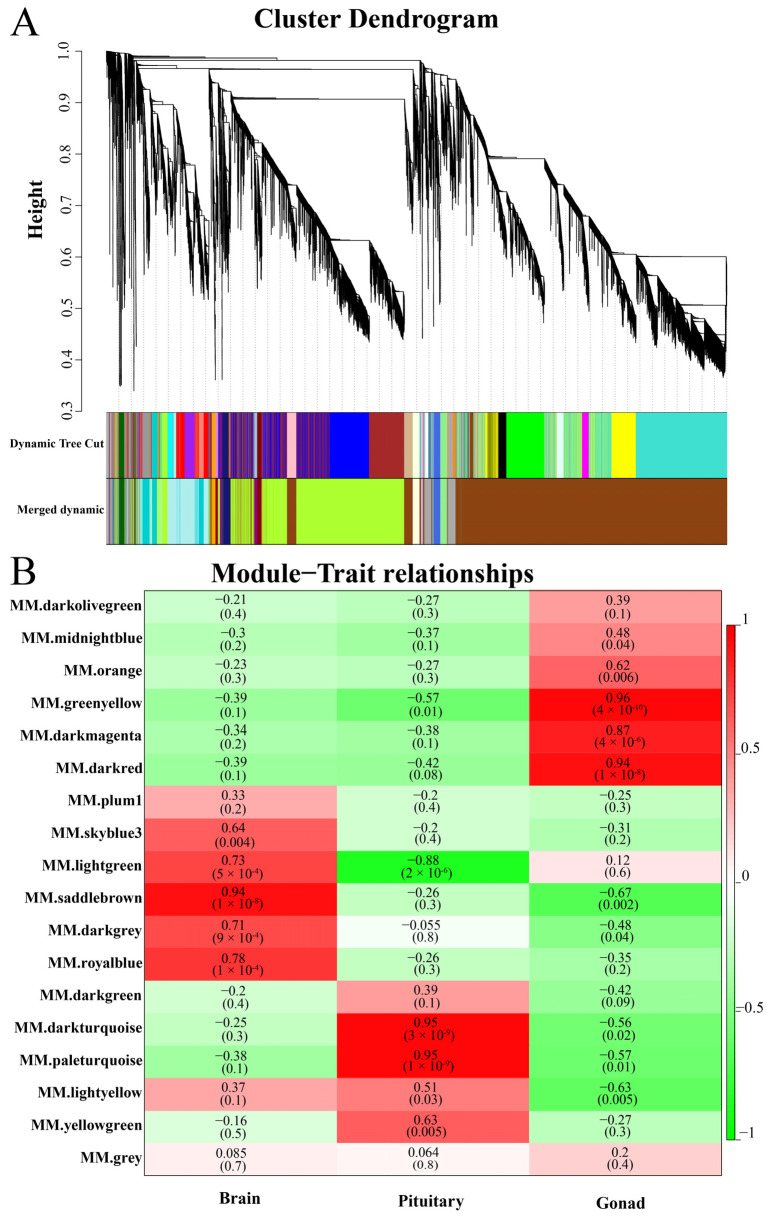
Weighted gene co-expression network analysis (WGCNA). (**A**) Clustering dendrogram of modules; and (**B**) heatmap of correlations between modules and traits. The modules with high correlation coefficients and *p* < 0.05 were considered significant trait-related modules. Green bars indicate negative correlations and red indicates positive correlations; the closer the absolute value is to 1, the higher is the correlation.

In total, 22,226 high-quality genes (FPKM value > 1) were imported into WGCNA. A gene cluster dendrogram was constructed according to the correlation of gene expression, and the genes with similar expression patterns were classified into the same module according to the clustering relationship (Figure 4A). Finally, 18 modules were obtained, of which the saddle brown module genes were the greatest in number (i.e., 10,812), and plum1 module genes were the least (i.e., 60) (Table S5). In addition, the target modules related to gonadal development were identified according to the correlation coefficient between gene expression patterns and sample traits. The saddlebrown, paleturquoise, and greenyellow modules were significantly positively correlated with gonadal development in the brain, pituitary, and gonad, respectively (Figure 4B). Although the paleturquoise module and darkturquoise module have the same the correlation, the paleturquoise module has a smaller *p*-value, and so we chose the paleturquoise module.

### 3.6. Functional Analysis of Target Modules

A KEGG analysis was performed on genes within the target modules to reveal the relationship between HPG axis tissues and gonadal development. The saddlebrown module was the target module in the brain related to gonadal development. The saddlebrown module exhibited significant enrichment of 35 KEGG pathways (*p* < 0.05), including the MAPK signaling pathway, ErbB signaling pathway, and oxytocin signaling pathway (Figure S4A). The paleturquoise module exhibited significant enrichment of 28 KEGG pathways (*p* < 0.05). This module was closely related to the pituitary and showed enrichment for pathways related to hormone synthesis, such as thyroid hormone synthesis, ovarian steroidogenesis, and estrogen signaling pathways (Figure S4B). The greenyellow module was identified as a target module in the gonad related to gonadal development, with 47 significantly enriched KEGG pathways (*p* < 0.05), including DNA replication, cell cycle, and Fanconi anemia pathways (Figure S4C).

### 3.7. Network Construction and Gene Identification

**Figure 5:**
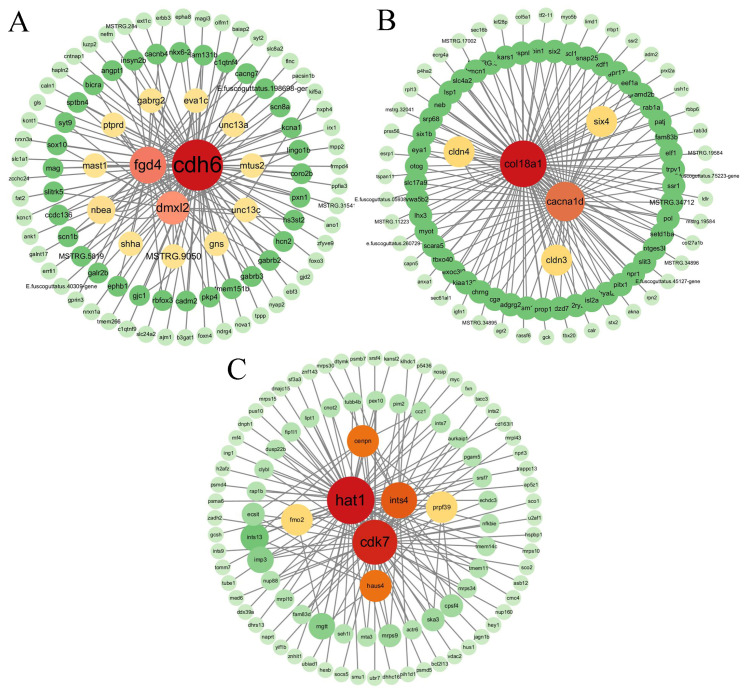
Gene co-expression network relationships in each target module. Networks were visualized based on (**A**) 98 genes from the saddlebrown module, (**B**) 98 genes from the paleturquoise module, and (**C**) 98 genes from the greenyellow module.

The genes with the first 10 MM (module membership) values in the saddlebrown, paleturquoise, and greenyellow modules were introduced into Cytoscape v3.9.1, and the first 150 pairs were selected to construct the co-expression network. The gene with the highest connectivity in each module was used as the hub gene. We identified *cdh6*, *col18a1* and *hat1* as hub genes in the saddlebrown, paleturquoise, and greenyellow modules, respectively (Figure 5).

### 3.8. qRT-PCR Validation

**Figure 6:**
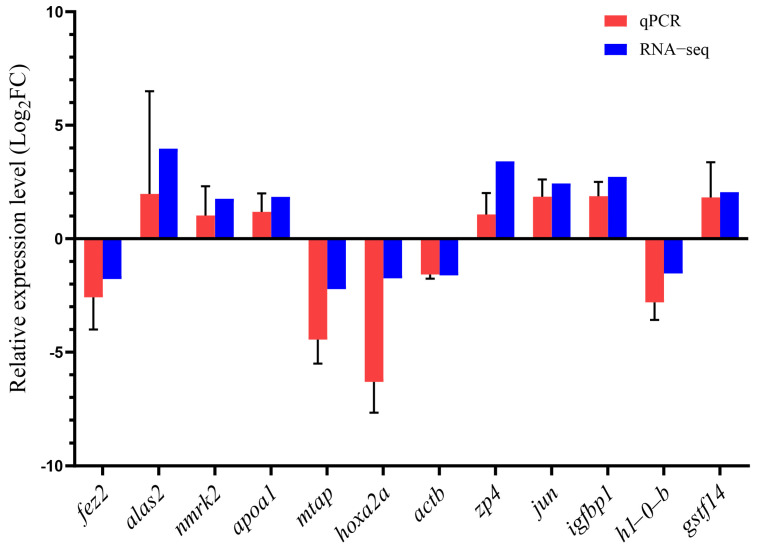
Quantitative real-time PCR (qRT-PCR) validation of transcriptome data. Expression fold change values for 12 genes in the tiger grouper and Jinhu grouper detected by qRT-PCR and RNA-Seq were calculated using the 2^−ΔΔCt^ method and FPKM (fragment per kilobase of transcript per million mapped reads), respectively. The log_2_ fold change values for RNA-Seq and qRT-PCR data are shown in blue and red, respectively. For qRT-PCR, values are indicated as means ± standard deviation (SD) (*n* = 3). β-actin was used as the internal reference.

Twelve genes were selected for qRT-PCR validation to confirm the accuracy of the RNA-seq data. These genes have expression patterns consistent with RNA-Seq, supporting the reliability of the transcriptome data (Figure 6).

## 4. Discussion

With the support of modern molecular technologies, significant advancements have been achieved in comprehending gonadal development in fish, especially the gene regulatory mechanisms underlying germ cell formation, gonadal differentiation, and sex determination [27]. The HPG axis regulates fish gonadal differentiation and development by synthesizing and secreting reproductive hormones [28]. In this study, we compared the transcriptomes of brain, pituitary, and gonadal tissues between the Jinhu grouper and tiger grouper at 24-months old, and 3034 DEGs were obtained. The differential expression analysis of genes in the HPG axis between the Jinhu grouper and tiger grouper provided insight into the molecular mechanism underlying gonadal growth retardation in the Jinhu grouper.

In the brain, significantly enriched pathways include ovarian steroidogenesis, the JAK-STAT signaling pathway, osteoclast differentiation, and other pathways related to cell growth and differentiation, as well as ovarian steroidogenesis and the biosynthesis of ovarian steroids. In this study, the expression of *nr4a1* in the Jinhu grouper was significantly lower than that in the tiger grouper It has been shown that *nr4a1* is involved in the regulation of hormone genes in the hypothalamic–pituitary–adrenal axis and HPG axis, including pro-opiomelanocortin (POMC) in the pituitary, 20α-HSD in the ovary, CYP11B2 and 3β-HSD in the adrenal gland, and CYP17 and 3β-HSD in the testis [29]. In *Rattus norvegicus*, *nr4a1* is expressed in growing and mature follicular cells; it can respond to changes in LH, thereby affecting ovulation [30]. *Nr4a1* and *StAR* have the same expression pattern in tissues such as gonad and adrenal, and are regulated by steroid hormone-related genes [31]. In addition, *nr4a1* can promote the transcription of *AR* promoter and promote the expression of AR protein, which plays an important role in the process of follicular maturation [32]. In this study, the down-regulation of *nr4a1* expression in the Jinhu grouper may be the reason for the low level of serum steroid hormones in the Jinhu grouper.

The pituitary translates hypothalamic regulatory signals into endocrine responses and participates in various physiological processes, encompassing metabolism and reproduction [33]. In the pituitary, a KEGG analysis of DEGs indicated significant enrichment of the NF-κB signaling pathway, oocyte meiosis, cholesterol metabolism, and other pathways related to ovarian development. The oocyte meiosis pathway contributes to oocyte growth, meiosis, and the fertilization/activation stage [34]. In the oocyte meiosis pathway, the expression levels of *pgr*, *ar*, *camk2a*, and other genes related to oocyte development in the tiger grouper were significantly higher than those in the Jinhu grouper. Progesterone functions via *pgr* during meiosis and regulates oocyte development and ovulation in various vertebrates [35]. In addition, the expression of *dmrta2* in the Jinhu grouper was significantly higher than that in the tiger grouper. *Dmrta2* (*dmrt5*) is a member of the Dmrt gene family, sharing a conserved DM domain. It has been found that *dmrta2* promotes adrenocorticotropin differentiation and inhibits gonadotropin differentiation via *tbx19* in zebrafish [36]. In this study, it is possible that the up-regulation of *dmrta2* and *tbx19* in the Jinhu grouper leads to a decrease in gonadotropin secretion and ovarian growth retardation.

In the gonad, DEGs were involved in various pathways, including metabolic pathways, the PI3K-Akt signaling pathway, and ovarian steroidogenesis. The PI3K-Akt signaling pathway is a key regulator of ovarian development [37]. Ovarian steroidogenesis and the estrogen signaling pathway are involved in the biosynthesis of sex hormones and have a direct impact on gonadal development or differentiation [38]. This study showed that *cyp19a1* was significantly down-regulated in the hybrid offspring, consistent with results for offspring of *Misgurnus anguillicaudatus* and *Paramisgurnus dabryanus* [39]. As one of the most highly conserved enzymes, *cyp19a1*-encoded aromatase converts androgens into estrogens and controls ovarian differentiation, development, and growth in bony fishes. It is often reported that the *cyp19a1* expression or activity and serum E2 levels vary with the sex change and ovarian development of fish [40,41,42], indicating that there is a positive correlation between *cyp19a1* and E2. It is speculated that low *cyp19a1* expression in hybrids contributes to the significantly lower level of serum E2 than that of female parents, as well as ovarian growth retardation.

Ovarian development in teleost fish is regulated by the HPG axis. In this study, based on transcriptome data for 18 brain, pituitary, and gonad samples from 24-month-old Jinhu groupers and tiger groupers, WGCNA was performed after filtering out genes with low expression. Three modules, saddlebrown, paleturquoise, and greenyellow, were significantly related to ovarian development. The genes in these three modules revealed significant enrichment for pathways related to ovarian development, such as the oxytocin signaling pathway in the saddlebrown module, prolactin signaling pathway in the paleturquoise module, and Fanconi anemia signaling pathway in the greenyellow module. Three hub genes in the three modules were obtained, namely *cdh6*, *col18a1*, and *hat1*.

*Cdh6* (K-cadherin) belongs to type II classical cadherin, and it facilitates cell adhesion primarily through homophilic interactions and participates in the morphogenesis of diverse tissues and organs [43]. *Cdh6* is a nodal protein that participates in stem cell genesis and development by interacting with signal transduction molecules [44]. In addition, given that *cdh6* gene expression is regulated by paired-box 8 (PAX8), a key lineage-specific transcriptional regulator of the development of kidney, ovarian, and thyroid tissues, it can be inferred that *cdh6* may function as a lineage-specific developmental regulator for these tissues [45]. *Col18a1* is a member of the collagen family genes. The *col18a1* gene encodes XVIIIA1 collagen and can bind to and regulate several molecules related to cell development and differentiation, including Wnt, TGF-β, and fibroblast growth factor family members [46,47,48]. Collagen is an important component of the ECM. In the process of oocyte maturation, cumulus cells are involved in gap junction, adhesion, and tight junction communication with oocytes through the ECM [49]. Cumulus cells participate in the proliferation and differentiation of oocytes through the ECM, and interact with closed oocytes through cross-region projection [50]. HAT1 was the first histone acetyltransferase found. In eukaryotes, the highly conserved HAT complex plays an important role in mitosis and meiosis [51]. Therefore, HATS is necessary for cell growth and differentiation. Abundant HAT1 holoenzymes were purified from *Xenopus* oocytes; levels were 10,000 times higher than those in somatic cells [52]. With the increase of maternal age, the expression of *hat1* in granulosa cells decreased significantly, indicating that *hat1* is highly correlated with oocyte maturation [53]. To sum up, the three hub genes identified in the three modules may play important roles in ovarian development in the Jinhu grouper.

## 5. Conclusions

Eighteen samples of 24-month-old Jinhu groupers and tiger groupers were used for a comparative transcriptome analysis of HPG axis tissues. In total, 771, 1630, and 1175 DEGs were obtained in brain, pituitary, and gonadal tissues, respectively. The DEGs were involved in various pathways, including ovarian steroidogenesis, the NF-κB signaling pathway, oocyte meiosis, cholesterol metabolism, and other signaling pathways related to cell growth and ovarian development. Furthermore, *nr4a1*, *pgr*, *dmrta2*, *tbx19*, and *cyp19a1* were identified as candidate genes related to gonadal retardation in the Jinhu grouper. Three modules related to ovarian development were obtained by WGCNA, and genes with high correlations, such as *cdh6*, *col18a1*, and *hat1*, were screened. This study provides additional insight into the molecular mechanism underlying ovarian stunting in grouper hybrids.

## Data Availability

The raw reads were submitted to the National Center for Biotechnology Information (NCBI) Sequence Read Archive (SRA) with accession number PRJNA955319.

## Supplementary Materials

The following supporting information can be downloaded at: https://www.mdpi.com/article/10.3390/genes15070929/s1, Figure S1: Gene Ontology (GO) enrichment analysis of brain (A), pituitary (B), and gonad (C) from Jinhu grouper (EFET) vs tiger grouper (EF); Figure S2: Kyoto Encyclopedia of Genes and Genomes (KEGG) enrichment analysis of brain, pituitary, and gonad from Jinhu grouper (EFET) vs tiger grouper (EF) (p-value); Figure S3: Kyoto Encyclopedia of Genes and Genomes (KEGG) enrichment analysis of profile 0 (A), profile 3 (B), profile 4 (C) (p-value); Figure S4: Kyoto Encyclopedia of Genes and Genomes (KEGG) enrichment analysis of saddlebrown module (A), paleturquoise module (B), and greenyellow module (C) (q-value).; Table S1: Sequences of specific primers for the quantitative real-time PCR (qRT-PCR); Table S2: The reads information in the transcriptomic analysis; Table S3: The quality control data in the transcriptomic analysis; Table S4: Statistics in mapping of transcriptome data to reference genome; Table S5: The gene number in each module; Table S6: A list of genes in each module.
