# Supplementary material for: Comparative Transcriptome Analysis of the Hypothalamic–Pituitary–Gonadal Axis of Jinhu Grouper (Epinephelus fuscoguttatus ♀ × Epinephelus tukula ♂) and Tiger Grouper (Epinephelus fuscoguttatus)
Source: Genes (Basel). 2024 Jul 16;15(7):929. doi: 10.3390/genes15070929 (PMC11275438; doi:10.3390/genes15070929)
Supplement: Supplementary file 1 [file genes-15-00929-s001.zip › Figure S1-4, Table S1-5.pdf]

**Supplementary materials for**

**Comparative transcriptome analysis of the hypothalamic–pituitary–gonadal axis of Jinhu grouper (*Epinephelus fuscoguttatus* ♀ × *Epinephelus tukula* ♂) and tiger grouper (*Epinephelus fuscoguttatus*)**

Fig. S1. Gene Ontology (GO) enrichment analysis of brain (A), pituitary (B), and gonad (C) from Jinhu grouper (EFET) vs tiger grouper (EF).

Fig. S2. Kyoto Encyclopedia of Genes and Genomes (KEGG) enrichment analysis of brain, pituitary, and gonad from Jinhu grouper (EFET) vs tiger grouper (EF) (*p*-value).

Fig. S3. Kyoto Encyclopedia of Genes and Genomes (KEGG) enrichment analysis of profile 0 (A), profile 3 (B), profile 4 (C) (*p*-value).

Fig. S4. Kyoto Encyclopedia of Genes and Genomes (KEGG) enrichment analysis of saddlebrown module (A), paleturquoise module (B), and greenyellow module (C) (*q*-value).

Table S1. Sequences of specific primers for the quantitative real-time PCR (qRT-PCR).

Table S2. The reads information in the transriptomic analysis.

Table S3. The quality control data in the transriptomic analysis.

Table S4. Statistics in mapping of transcriptome data to reference genome.

Table S5. The gene number in each module.

Fig. S1. Gene Ontology (GO) enrichment analysis of brain (A), pituitary (B), and gonad (C) from Jinhu grouper (EFET) vs tiger grouper (EF).

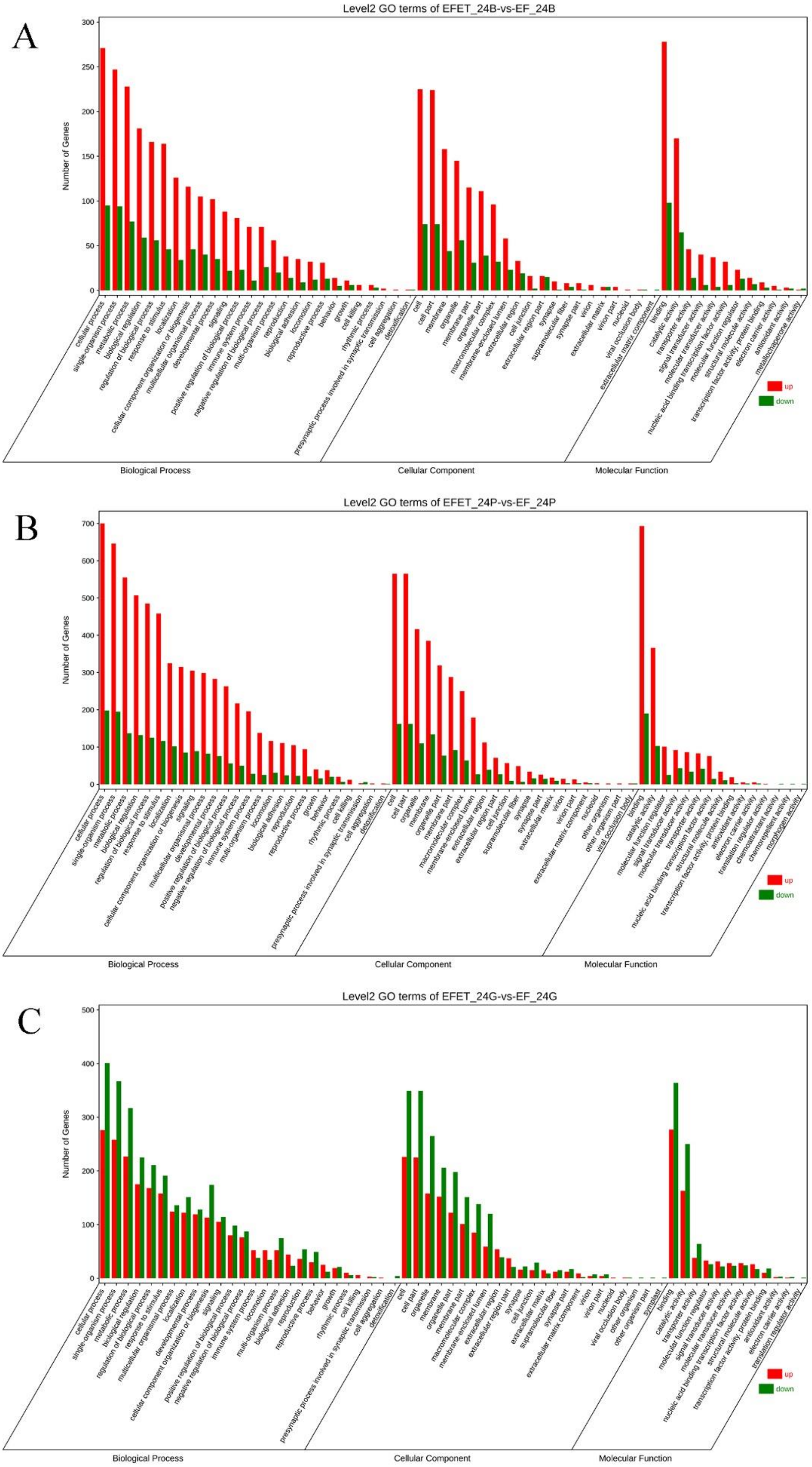

Fig. S2. Kyoto Encyclopedia of Genes and Genomes (KEGG) enrichment analysis of brain, pituitary, and gonad from Jinhu grouper (EFET) vs tiger grouper (EF) (*p*-value).

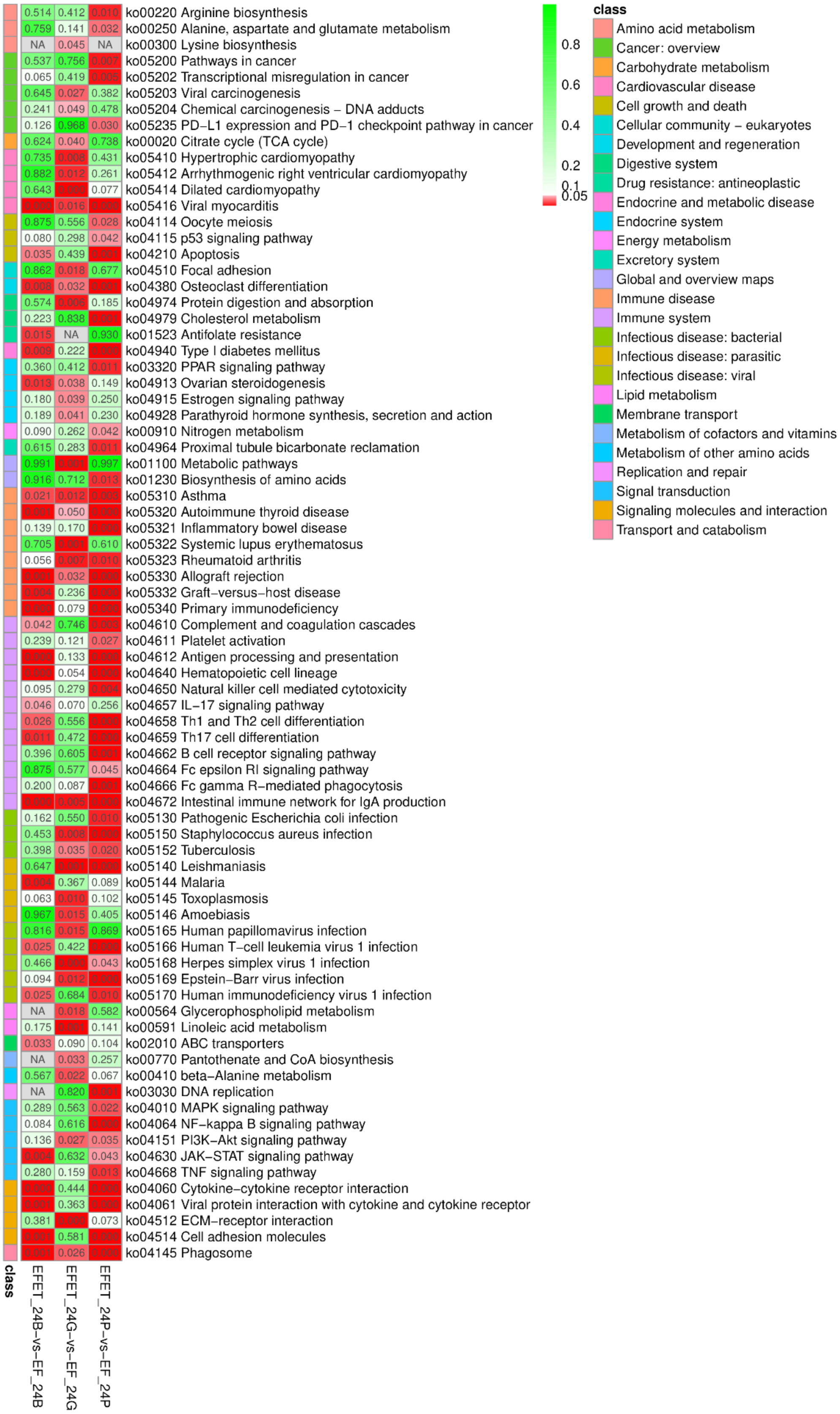

Fig. S3. Kyoto Encyclopedia of Genes and Genomes (KEGG) enrichment analysis of profile 0 (A), profile 3 (B), profile 4 (C) (*p*-value).

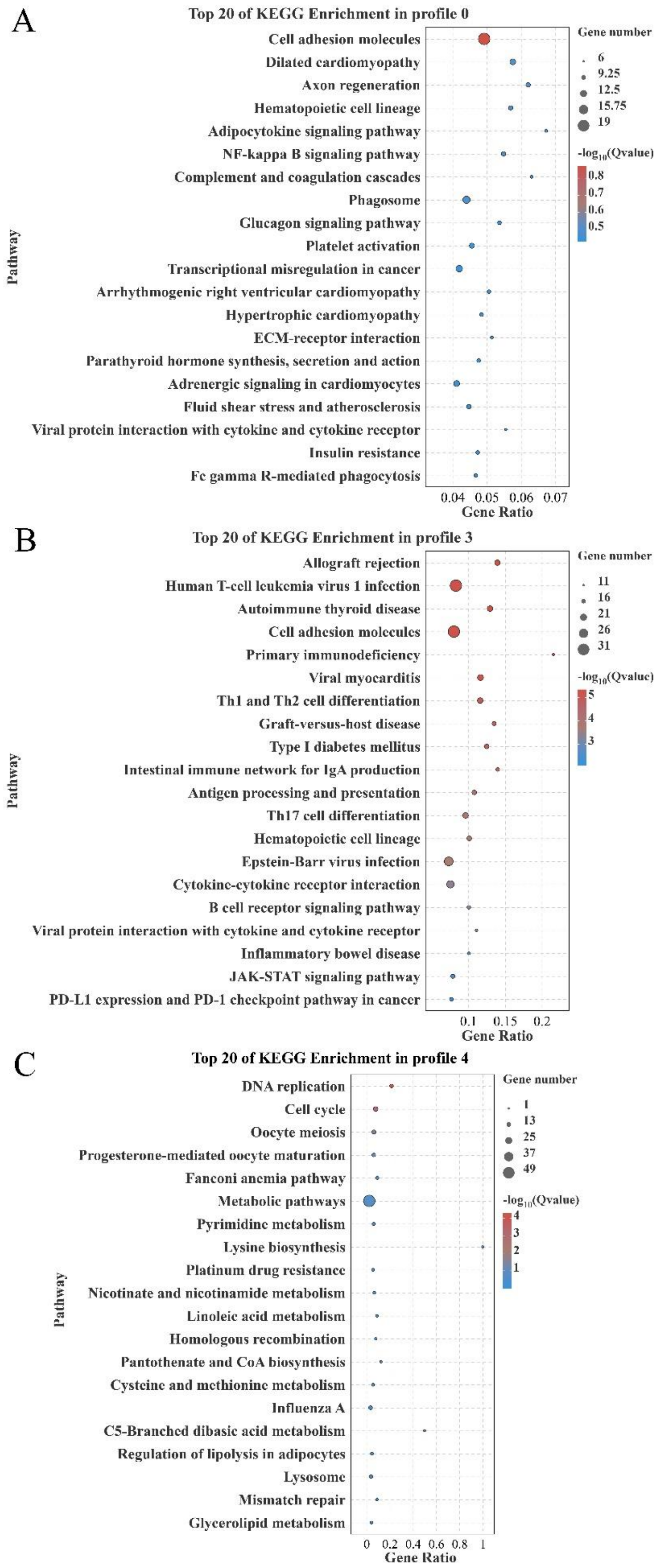

Fig. S4. Kyoto Encyclopedia of Genes and Genomes (KEGG) enrichment analysis of saddlebrown module (A), paleturquoise module (B), and greenyellow module (C) (*q*-value).

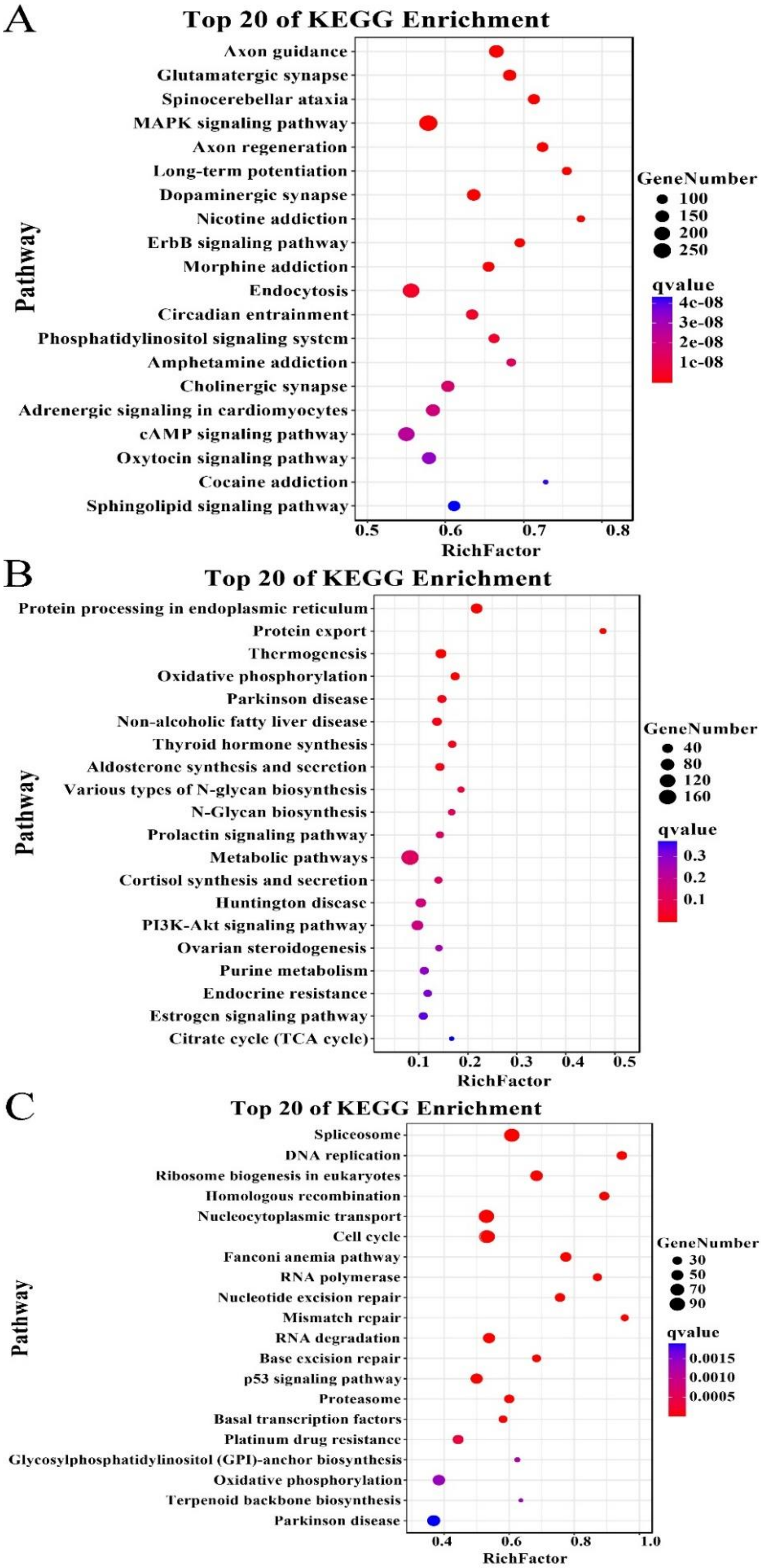

Table S1. Sequences of specific primers for the quantitative real-time PCR (qRT-PCR).

| Gene symbol | Forward Primer (5'→3') | Reverse Primer (5'→3') |
|---|---|---|
| *fez2* | ACAAAACCGACAGAAGGAGCA | CAGAGGAGATTCCCTCCACG |
| *alas2* | TCTACGTCCAGGCCATCAA | CCACACCTCCACCAGTTTCT |
| *nmrk2* | GGCGTAACTAACGGAGGGA | GATCTTGGGGCTTGAAGAAG |
| *apoa1* | TCTTGCTCTCGCCCTTCT | TTGGCACTGTCCTTCACCT |
| *mtap* | AGGGAAGGACCGAGCGTTAT | CCATGCCTTGCAAGAAGCAC |
| *hoxa2a* | CGAGAGAGCGGTTTTATCAA | AGGAATCAGTGTCGGGCGT |
| *actb* | CTGCTGACTGAGGCTCCACT | TCACCAGAGTCCAGCACAATA |
| *zp4* | TAGCTCGTTCTACACTGCGG | ATTGGGTGTAGATGTGGCCC |
| *jun* | GACCCCGACACAGTTCATGT | ATTAGTTGTGCCCGGCATGT |
| *igfbp1* | CAACTGTGACAAACACGGGC | GGCAAGTCGGTCGAACCTAA |
| *h1-0-b* | GACAGCCAAGCCCAAGAAAC | TGTCGCCCACCTTGTAGTTC |
| *gstf14* | GTCCCAGAGGGAGAGAGACA | CACATCAGCCAGTGAAAAGC |
| *β-actin* | AGTTGTTGGGCGTTTGGTC | GTGGATCAGCAAGCAGGAGTAC |

Table S2. The reads information in the transriptomic analysis.

| Sample | Raw reads | Clean reads (%) | Adapter (%) | Low quality (%) | polyA (%) | N (%) |
|---|---|---|---|---|---|---|
| EFET_24B1 | 54261686 | 54034718 (99.58%) | 24148 (0.04%) | 201910 (0.37%) | 0 (0.00%) | 910 (0.00%) |
| EFET_24B2 | 50817230 | 50600900 (99.57%) | 22106 (0.04%) | 193272 (0.38%) | 0 (0.00%) | 952 (0.00%) |
| EFET_24B3 | 52253326 | 52016040 (99.55%) | 25370 (0.05%) | 211092 (0.40%) | 0 (0.00%) | 824 (0.00%) |
| EFET_24G1 | 53776710 | 53542412 (99.56%) | 32194 (0.06%) | 202104 (0.38%) | 0 (0.00%) | 0 (0.00%) |
| EFET_24G2 | 63335786 | 63033870 (99.52%) | 40360 (0.06%) | 261556 (0.41%) | 0 (0.00%) | 0 (0.00%) |
| EFET_24G3 | 67507892 | 67263386 (99.64%) | 40410 (0.06%) | 204096 (0.30%) | 0 (0.00%) | 0 (0.00%) |
| EFET_24P1 | 40276342 | 40079044 (99.51%) | 16140 (0.04%) | 181158 (0.45%) | 0 (0.00%) | 0 (0.00%) |
| EFET_24P2 | 46860988 | 46700586 (99.66%) | 17212 (0.04%) | 143190 (0.31%) | 0 (0.00%) | 0 (0.00%) |
| EFET_24P3 | 49979844 | 49811040 (99.66%) | 14544 (0.03%) | 154260 (0.31%) | 0 (0.00%) | 0 (0.00%) |
| EF_24B1 | 45325640 | 45122012 (99.55%) | 18572 (0.04%) | 185056 (0.41%) | 0 (0.00%) | 0 (0.00%) |
| EF_24B2 | 39361762 | 39203952 (99.60%) | 19092 (0.05%) | 137304 (0.35%) | 0 (0.00%) | 1414 (0.00%) |
| EF_24B3 | 42721398 | 42549666 (99.60%) | 22090 (0.05%) | 148194 (0.35%) | 0 (0.00%) | 1448 (0.00%) |
| EF_24G1 | 52106000 | 51906922 (99.62%) | 23586 (0.05%) | 175492 (0.34%) | 0 (0.00%) | 0 (0.00%) |
| EF_24G2 | 36981238 | 36814376 (99.55%) | 26248 (0.07%) | 139590 (0.38%) | 0 (0.00%) | 1024 (0.00%) |
| EF_24G3 | 51270624 | 51063610 (99.60%) | 30308 (0.06%) | 175800 (0.34%) | 0 (0.00%) | 906 (0.00%) |
| EF_24P1 | 37916662 | 37731946 (99.51%) | 15726 (0.04%) | 168990 (0.45%) | 0 (0.00%) | 0 (0.00%) |
| EF_24P2 | 44941328 | 44740408 (99.55%) | 17152 (0.04%) | 183768 (0.41%) | 0 (0.00%) | 0 (0.00%) |
| EF_24P3 | 45762106 | 45564534 (99.57%) | 18040 (0.04%) | 179532 (0.39%) | 0 (0.00%) | 0 (0.00%) |

Table S3. The quality control data in the transriptomic analysis.

| Sample | Raw data (bp) | Clean data (bp) | Q20 (%) | Q30 (%) | N (%) | GC (%) |
|---|---|---|---|---|---|---|
| EFET_24B1 | 8139252900 | 8071620029 | 7884762236 (97.69%) | 7543461379 (93.46%) | 32278 (0.00%) | 3693383917 (45.76%) |
| EFET_24B2 | 7622584500 | 7551013799 | 7364080018 (97.52%) | 7025048720 (93.03%) | 30851 (0.00%) | 3475309072 (46.02%) |
| EFET_24B3 | 7837998900 | 7759123651 | 7576452425 (97.65%) | 7242213537 (93.34%) | 30955 (0.00%) | 3554289075 (45.81%) |
| EFET_24G1 | 8066506500 | 7990577896 | 7734706465 (96.80%) | 7306357346 (91.44%) | 21982 (0.00%) | 4045799817 (50.63%) |
| EFET_24G2 | 9500367900 | 9394335142 | 9046302263 (96.30%) | 8497882614 (90.46%) | 25553 (0.00%) | 4787841792 (50.97%) |
| EFET_24G3 | 10126183800 | 10036607249 | 9722463722 (96.87%) | 9169446808 (91.36%) | 19511 (0.00%) | 5138167636 (51.19%) |
| EFET_24P1 | 6041451300 | 5984996390 | 5805155586 (97.00%) | 5493582500 (91.79%) | 16258 (0.00%) | 2882987871 (48.17%) |
| EFET_24P2 | 7029148200 | 6978415807 | 6786398318 (97.25%) | 6423813739 (92.05%) | 13398 (0.00%) | 3366415270 (48.24%) |
| EFET_24P3 | 7496976600 | 7448443212 | 7244262264 (97.26%) | 6858939636 (92.09%) | 14524 (0.00%) | 3622539737 (48.63%) |
| EF_24B1 | 6798846000 | 6736763978 | 6524975420 (96.86%) | 6162107220 (91.47%) | 18320 (0.00%) | 3122626562 (46.35%) |
| EF_24B2 | 5904264300 | 5848030591 | 5709249884 (97.63%) | 5448476780 (93.17%) | 30395 (0.00%) | 2689385055 (45.99%) |
| EF_24B3 | 6408209700 | 6353779764 | 6181854729 (97.29%) | 5873988409 (92.45%) | 32450 (0.00%) | 2934491407 (46.18%) |
| EF_24G1 | 7815900000 | 7760242039 | 7523698700 (96.95%) | 7112743435 (91.66%) | 21327 (0.00%) | 3925326689 (50.58%) |
| EF_24G2 | 5547185700 | 5493077123 | 5308456982 (96.64%) | 5011767907 (91.24%) | 25916 (0.00%) | 2777316994 (50.56%) |
| EF_24G3 | 7690593600 | 7626477549 | 7414520017 (97.22%) | 7051338524 (92.46%) | 31598 (0.00%) | 3858873526 (50.60%) |
| EF_24P1 | 5687499300 | 5639464429 | 5489529600 (97.34%) | 5211673537 (92.41%) | 15347 (0.00%) | 2645482753 (46.91%) |
| EF_24P2 | 6741199200 | 6682127961 | 6486457015 (97.07%) | 6137460878 (91.85%) | 18099 (0.00%) | 3169098701 (47.43%) |
| EF_24P3 | 6864315900 | 6807504239 | 6619004661 (97.23%) | 6276391150 (92.20%) | 18483 (0.00%) | 3207060547 (47.11%) |

Table S4. Statistics in mapping of transcriptome data to reference genome.

| Sample | Clean_reads | rRNA unmapped_reads (%) | Unmapped (%) | Unique_mapped (%) | Multiple_mapped (%) | Total_mapped (%) |
|---|---|---|---|---|---|---|
| EFET_24B1 | 54034718 | 53815800 (99.59%) | 10558093 (19.62%) | 42645922 (79.24%) | 611785 (1.14%) | 43257707 (80.38%) |
| EFET_24B2 | 50600900 | 50423750 (99.65%) | 9190179 (18.23%) | 40632112 (80.58%) | 601459 (1.19%) | 41233571 (81.77%) |
| EFET_24B3 | 52016040 | 51850450 (99.68%) | 9336874 (18.01%) | 41905999 (80.82%) | 607577 (1.17%) | 42513576 (81.99%) |
| EFET_24G1 | 53542412 | 53528662 (99.97%) | 6026698 (11.26%) | 44691949 (83.49%) | 2810015 (5.25%) | 47501964 (88.74%) |
| EFET_24G2 | 63033870 | 63015492 (99.97%) | 7563245 (12.00%) | 52080137 (82.65%) | 3372110 (5.35%) | 55452247 (88.00%) |
| EFET_24G3 | 67263386 | 67241954 (99.97%) | 7944921 (11.82%) | 55123449 (81.98%) | 4173584 (6.21%) | 59297033 (88.18%) |
| EFET_24P1 | 40079044 | 40033886 (99.89%) | 6159982 (15.39%) | 32825771 (81.99%) | 1048133 (2.62%) | 33873904 (84.61%) |
| EFET_24P2 | 46700586 | 46660546 (99.91%) | 6812375 (14.60%) | 38776309 (83.10%) | 1071862 (2.30%) | 39848171 (85.40%) |
| EFET_24P3 | 49811040 | 49768894 (99.92%) | 7681380 (15.43%) | 40705580 (81.79%) | 1381934 (2.78%) | 42087514 (84.57%) |
| EF_24B1 | 45122012 | 44983536 (99.69%) | 4551787 (10.12%) | 39829242 (88.54%) | 602507 (1.34%) | 40431749 (89.88%) |
| EF_24B2 | 39203952 | 39100294 (99.74%) | 3473191 (8.88%) | 35160238 (89.92%) | 466865 (1.19%) | 35627103 (91.12%) |
| EF_24B3 | 42549666 | 42450940 (99.77%) | 3409573 (8.03%) | 38489630 (90.67%) | 551737 (1.30%) | 39041367 (91.97%) |
| EF_24G1 | 51906922 | 51891516 (99.97%) | 1755358 (3.38%) | 47050559 (90.67%) | 3085599 (5.95%) | 50136158 (96.62%) |
| EF_24G2 | 36814376 | 36797584 (99.95%) | 1549951 (4.21%) | 32716622 (88.91%) | 2531011 (6.88%) | 35247633 (95.79%) |
| EF_24G3 | 51063610 | 51048854 (99.97%) | 1804494 (3.53%) | 45914139 (89.94%) | 3330221 (6.52%) | 49244360 (96.47%) |
| EF_24P1 | 37731946 | 37703712 (99.93%) | 2162792 (5.74%) | 34769963 (92.22%) | 770957 (2.04%) | 35540920 (94.26%) |
| EF_24P2 | 44740408 | 44714654 (99.94%) | 2808172 (6.28%) | 40738937 (91.11%) | 1167545 (2.61%) | 41906482 (93.72%) |
| EF_24P3 | 45564534 | 45527366 (99.92%) | 2540198 (5.58%) | 42001035 (92.25%) | 986133 (2.17%) | 42987168 (94.42%) |

Note: The reference genome is the known tiger grouper (*Epinephelus fuscoguttatus*) genome in the National Center for Biotechnology Information (https://ncbi.nlm.nih.gov/bioproject/PRJDB9224/).

Table S5. The gene number in each module.

| Module | Gene number |
|---|---|
| saddlebrown | 10812 |
| greenyellow | 6008 |
| paleturquoise | 1595 |
| darkturquoise | 769 |
| darkgrey | 591 |
| midnightblue | 369 |
| darkred | 315 |
| lightgreen | 258 |
| lightyellow | 255 |
| royalblue | 240 |
| darkmagenta | 216 |
| darkgreen | 205 |
| grey | 155 |
| orange | 153 |
| darkolivegreen | 80 |
| yellowgreen | 80 |
| skyblue3 | 74 |
| plum1 | 60 |
